# Taste aversion and adverse events contribute equally to OIT discontinuation in children: A real‐world multi‐allergen analysis

**DOI:** 10.1111/pai.70482

**Published:** 2026-09-16

**Authors:** Breiding Maria, Höfer Veronika, Boucq Camille, Trück Johannes

**Affiliations:** ^1^ Division of Allergy University Children's Hospital and Children's Research Center, University of Zurich (UZH) Zurich Switzerland

**Keywords:** adverse effects, child, desensitization, food hypersensitivity, oral immunotherapy, taste aversion, treatment adherence, treatment discontinuation

## Abstract

**Background:**

Although oral immunotherapy (OIT) is a promising disease‐modifying treatment, its real‐world effectiveness is limited by the treatment burden and premature cessation. Comparative data on OIT discontinuation across allergens and non‐immunological barriers remain scarce. This study investigated the rates, reasons, and timing of discontinuation in a pediatric multi‐allergen cohort and identified patient phenotypes.

**Methods:**

In this single‐center observational study, pediatric patients initiating OIT between July 2022 and March 2026 were prospectively enrolled. Reasons for discontinuation were documented at cessation and supplemented by chart review. Causes were categorized as adverse events (AE) or non‐AE factors (taste aversion, logistical barriers, anxiety/distress). Baseline characteristics were compared, and hierarchical clustering identified phenotypes.

**Results:**

Among 406 OIT courses in 307 patients, 61 (15%) were discontinued. Rates were highest for milk and lowest for cashew and sesame. AE and taste aversion were the most frequent reasons for discontinuation (41% (*n* = 25) and 39% (*n* = 24), respectively), followed by logistical barriers (11%, *n* = 7) and anxiety/distress (8%, *n* = 5). AE‐related discontinuation occurred earlier in the treatment, whereas taste aversion predominated during maintenance and across ages. Patients discontinuing due to AE had higher baseline sIgE. Multi‐food OIT was not associated with increased discontinuation. Exploratory cluster analysis identified three phenotypes; the high‐risk cluster (high sIgE, prior severe reactions, low reactive dose) showed taste aversion as the leading cause of discontinuation.

**Conclusion:**

Discontinuation in this real‐world cohort was driven equally by AE and behavioral barriers. Taste aversion emerged as a key determinant of adherence, including in younger patients. Improving OIT success requires patient‐centered strategies addressing both immunological and practical barriers.

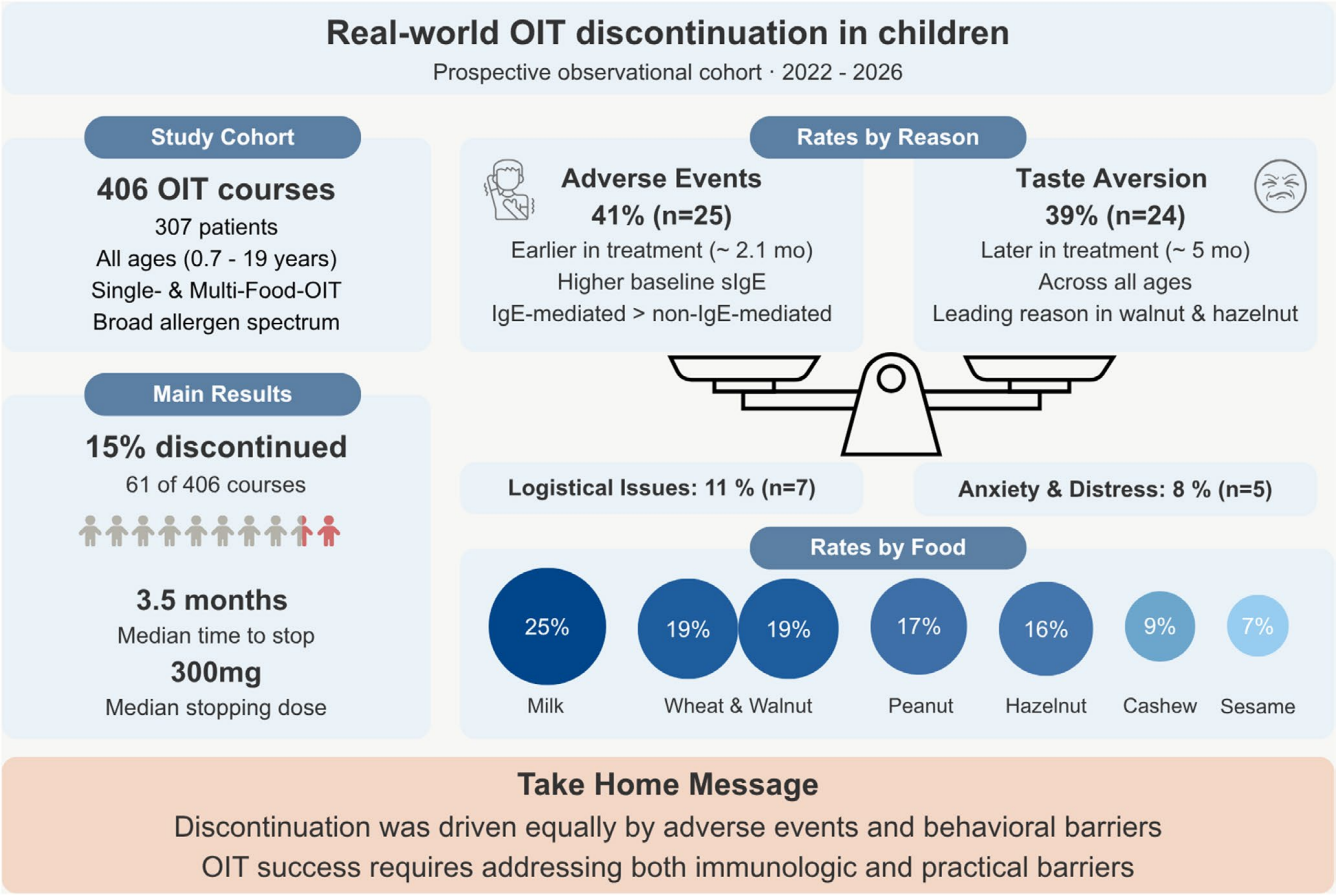

## Graphical abstract

Overview of discontinuation in a prospective real‐world pediatric OIT cohort, illustrating overall discontinuation, timing and dose at treatment cessation, clinical patterns associated with different reasons for discontinuation, and variation in discontinuation rates across individual food allergens.

**Abbreviations**
AE: Adverse events
EoE: Eosinophilic Esophagitis
oFASS‐5: Ordinal Food Allergy Severity Score‐5
OFC: Oral Food Challenge
OIT: Oral Immunotherapy
RD: Reactive Dose
sIgE: specific IgE

**Key message**

In this real‐world multi‐allergen pediatric cohort, 15% of OIT courses were discontinued, with substantial variation across allergens. Taste aversion and adverse events were the most frequent reasons for discontinuation. Whereas adverse event‐related discontinuation occurred earlier, taste aversion was more common during the maintenance phase and also affected younger patients. These findings underline the importance of long‐term follow‐up and of addressing both immunological and non‐immunological barriers in clinical practice.

## 1 INTRODUCTION

Food allergy is a common and increasing chronic disease in children, with an estimated prevalence of approximately 5%.^1^ It is the leading cause of anaphylaxis in childhood and is associated with a persistent risk of accidental exposure and reduced health‐related quality of life.^2^, ^3^, ^4^ Management has traditionally relied on strict allergen avoidance, an approach that addresses immediate safety but does not modify the underlying disease.

Oral immunotherapy (OIT) has emerged as a disease‐modifying alternative and is increasingly implemented in clinical practice worldwide. Since 2017, the European Academy of Allergy and Clinical Immunology (EAACI) has recommended OIT for selected patients with peanut, milk, and egg allergy, with updated guidance published in 2025.^5^ The primary goal is to increase the individual reaction threshold and thereby reduce the risk of severe reactions upon accidental exposure. Treatment outcomes are commonly categorized as desensitization, defined as a raised threshold during ongoing therapy, and sustained unresponsiveness, referring to a persistently raised threshold after therapy discontinuation. While sustained unresponsiveness is achieved less frequently, desensitization occurs in a substantial proportion of treated patients.^6^, ^7^, ^8^, ^9^

Despite these promising outcomes, real‐world implementation of OIT remains challenging. Reported discontinuation rates range from 4.4% to 43%.^7^, ^9^, ^10^, ^11^, ^12^, ^13^, ^14^ IgE‐mediated adverse events are common, ranging from mild local to severe systemic reactions,^15^, ^16^, ^17^ while non‐IgE‐mediated complications, including gastrointestinal symptoms and eosinophilic esophagitis (EoE), may also necessitate treatment cessation.^18^, ^19^ Beyond immunological factors, behavioral and practical barriers such as taste aversion, dose refusal, and anticipatory anxiety further limit long‐term adherence.^20^, ^21^ Early discontinuation has direct consequences for patients by preventing them from realizing treatment benefits and indirect costs through inefficient use of specialist resources.

Available data on discontinuation remain largely restricted to individual allergens, predominantly peanut, egg, and milk, with limited comparative evidence across a broader spectrum of food allergens treated in real‐world practice.^9^, ^15^, ^18^, ^20^, ^22^, ^23^ The primary objective of this study was to characterize reasons for OIT discontinuation across multiple allergens in a prospective real‐world cohort. Secondary objectives were to identify clinical and immunological factors associated with specific discontinuation patterns and to define distinct patient phenotypes. By delineating risk profiles linked to different barriers, we aim to inform more tailored monitoring and support strategies to improve long‐term treatment adherence.

## 2 METHODS

### 2.1 Study design and population

We conducted a single‐center observational cohort study at the University Children's Hospital Zurich. All patients recorded in our institutional OIT database who initiated treatment for an IgE‐mediated food allergy between July 1, 2022 and March 31, 2026, were included. Overall, 325 patients initiated 430 OIT courses involving 14 different food allergens. Both single‐food and multi‐food OIT were offered as part of routine clinical care. Multi‐food OIT was defined as OIT to more than one food allergen in the same patient, whether initiated concurrently or sequentially.

Patients initiating OIT during the study period constituted the reference population. Those who permanently discontinued at least one OIT course were defined as the discontinuation group; patients remaining on all initiated therapies at data cut‐off of March 31, 2026, were classified as the ongoing group.

Inclusion required documented informed consent for use of clinical data. No formal age restriction was applied.

### 2.2 OIT protocol

OIT was offered for peanut, tree nuts, sesame, milk, egg, wheat, and soy to patients with confirmed IgE‐mediated food allergy, following shared decision‐making and in the absence of contraindications. An open‐label oral food challenge (OFC) was routinely performed at baseline to confirm the diagnosis and determine the individual reactive dose (RD), which guided the starting dose.

The protocol comprised an up‐dosing phase targeting a maintenance dose of 300–1000 mg protein, with higher target doses for wheat, individualized according to tolerability and treatment goals, followed by an approximately 18‐month maintenance phase. A final OFC was performed to assess desensitization. Patients passing the final challenge were advised to continue regular allergen intake at least twice weekly. Sustained unresponsiveness was not assessed.

Protocol adaptations, including dose reductions or extended intervals, were implemented in patients with recurrent adverse reactions. Adjunctive omalizumab therapy was used in selected cases. Patients developing symptoms suggestive of eosinophilic esophagitis (EoE) were referred for gastroenterological evaluation, including endoscopy and biopsy where indicated.

Patients and caregivers received direct contact details of the allergy team, including an on‐call telephone number providing physician access outside regular clinic hours and at weekends. Families were encouraged to contact the team regarding adverse reactions, intercurrent illness, dosing difficulties, or adherence concerns. After completion of the up‐dosing phase, follow‐up visits were scheduled approximately every 6 months and, after successful desensitization, annually.

### 2.3 Classification of reasons for discontinuation

Discontinuation was defined as permanent cessation of OIT for medical or personal reasons. Temporary interruptions of less than 3 months were not classified as discontinuation. Patients restarting OIT after a gap of 3 months or more were recorded as new treatment attempts.

Reasons for discontinuation were assigned to predefined categories based on the primary documented cause. Where multiple contributing factors were present, the factor considered to have primarily driven the final decision to stop OIT was used for classification. Adverse events (AE) were classified as immediate (onset ≤2 h post‐consumption, presumed IgE‐mediated) or non–immediate (onset >2 h). Immediate reactions were graded using the validated ordinal Food Allergy Severity Score (oFASS‐5),^24^ with grades 4 and 5 classified as severe reactions. Non–immediate reactions were sub‐categorized as biopsy‐confirmed EoE, EoE‐like symptoms without endoscopic or histological confirmation, other gastrointestinal symptoms, or other symptoms.

Personal reasons were classified into three mutually exclusive categories:

- Taste aversion: rejection of the allergen's organoleptic properties (flavor, smell, or texture), without fear of an allergic reaction being the primary driver.
- Logistical barriers: practical impediments to protocol adherence (e.g., travel, scheduling constraints), including cases where perceived treatment burden outweighed expected benefit. Low motivation was included when treatment burden outweighed perceived benefit, rather than because of sensory aversion or anxiety.
- Anxiety and distress: anticipatory anxiety or acute emotional distress related to OIT or dosing, fear of possible allergic reactions.

A detailed overview of the documented circumstances underlying each discontinuation category is provided in Table S1. Socioeconomic factors such as financial constraints or lack of insurance coverage were not reported as primary reasons for discontinuation in this cohort.

### 2.4 Data collection

Clinical data were prospectively entered into a standardized electronic case report form in REDCap (Research Electronic Data Capture platform). OFC characteristics (reactive dose, reaction severity graded using the oFASS‐5, and clinical objective) were documented using a structured clinical checklist as previously described.^25^ A history of severe allergic reactions before OIT initiation was defined as a previous reaction of grade III or higher according to the H. L. Müller classification, which is routinely used for clinical documentation in Switzerland.^26^ For patients who discontinued OIT, the primary reason for cessation was assigned based on prospective clinical documentation and patient or caregiver report, supplemented by structured retrospective chart review where necessary.

Baseline immunological profiling included allergen‐specific IgE (sIgE) to both allergen extract and the major allergen component for risk stratification (Ara h 2, Cor a 14, Jug r 1, Ana o 3, Bos d 8/Casein, Ses i 1, Gly m 5, Gliadin, Gal d 1/Ovomucoid).

### 2.5 Statistical analysis

All analyses were performed in R (version 4.5.2, R Foundation for Statistical Computing, Vienna, Austria). Patient characteristics and outcomes were summarized using descriptive statistics. Subgroup analyses by allergen were restricted to groups with at least ten initiated OIT courses. Whether the analysis was conducted on OIT‐course level or patient‐level was decided according to the nature of the variable and research question. Patient‐level characteristics that do not vary between OIT courses, including sex and atopic comorbidities, were analyzed once per patient. Treatment‐specific characteristics were analyzed at the OIT‐course level. Consequently, patients receiving OIT for more than one food could contribute more than one observation to course‐level analyses. Continuous variables were compared using the Mann–Whitney *U* test or Kruskal–Wallis test, and categorical variables using Pearson's chi‐squared test or Fisher's exact test, as appropriate. A two‐sided *p*‐value of <.05 was considered statistically significant.

Cumulative incidence functions were estimated using a competing‐risks approach, with time calculated from OIT initiation until discontinuation or censoring at the study cut‐off date of March 31, 2026.

To identify distinct clinical phenotypes among patients who discontinued OIT, exploratory hierarchical cluster analysis was performed using five baseline variables selected a priori based on clinical relevance and prior literature: age, log‐transformed food‐specific IgE, asthma status, history of severe reactions, and RD at baseline OFC. Analysis was conducted at the patient level, with one observation per patient. Cases with missing baseline data were excluded. Gower distance was used to handle mixed data types, and clustering was performed using Ward's minimum variance method (Ward.D2). Cluster stability was assessed by bootstrap resampling (B = 1000); a Jaccard index >0.75 was considered indicative of a stable cluster. Cluster analyses were performed using the cluster and fpc packages.

### 2.6 Ethics

The study was approved by the Cantonal Ethics Committee of Zurich (BASEC 2024–00666) and conducted in accordance with the Declaration of Helsinki. All patients or their legal guardians provided written informed consent for the use of clinical data.

## 3 RESULTS

### 3.1 Study population

**FIGURE 1:**
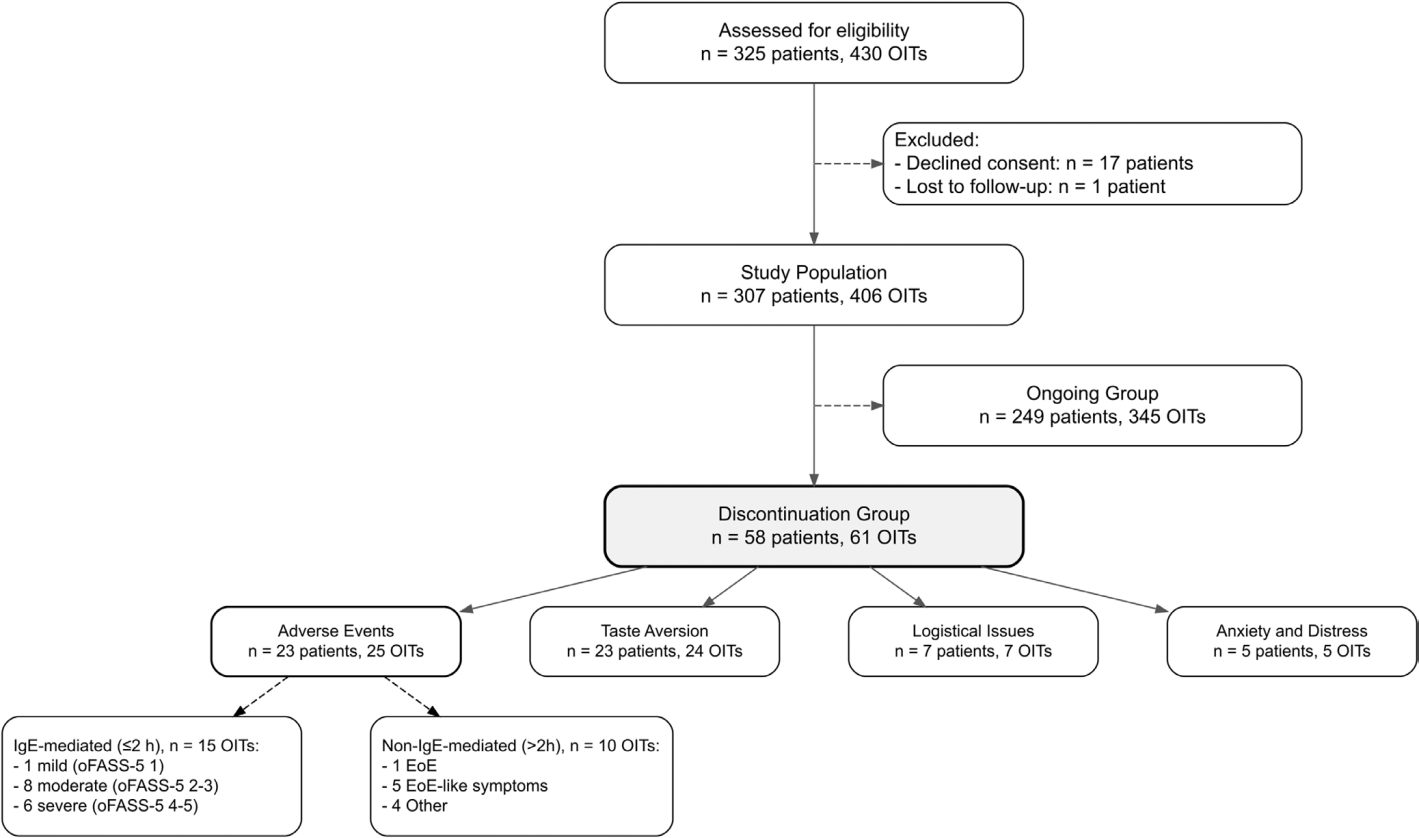
Flowchart of Patient Selection and Reasons for OIT discontinuation. EoE, eosinophilic esophagitis; oFASS‐5, ordinal Food Allergy Severity Scale; OIT, oral immunotherapy.

**FIGURE 2:**
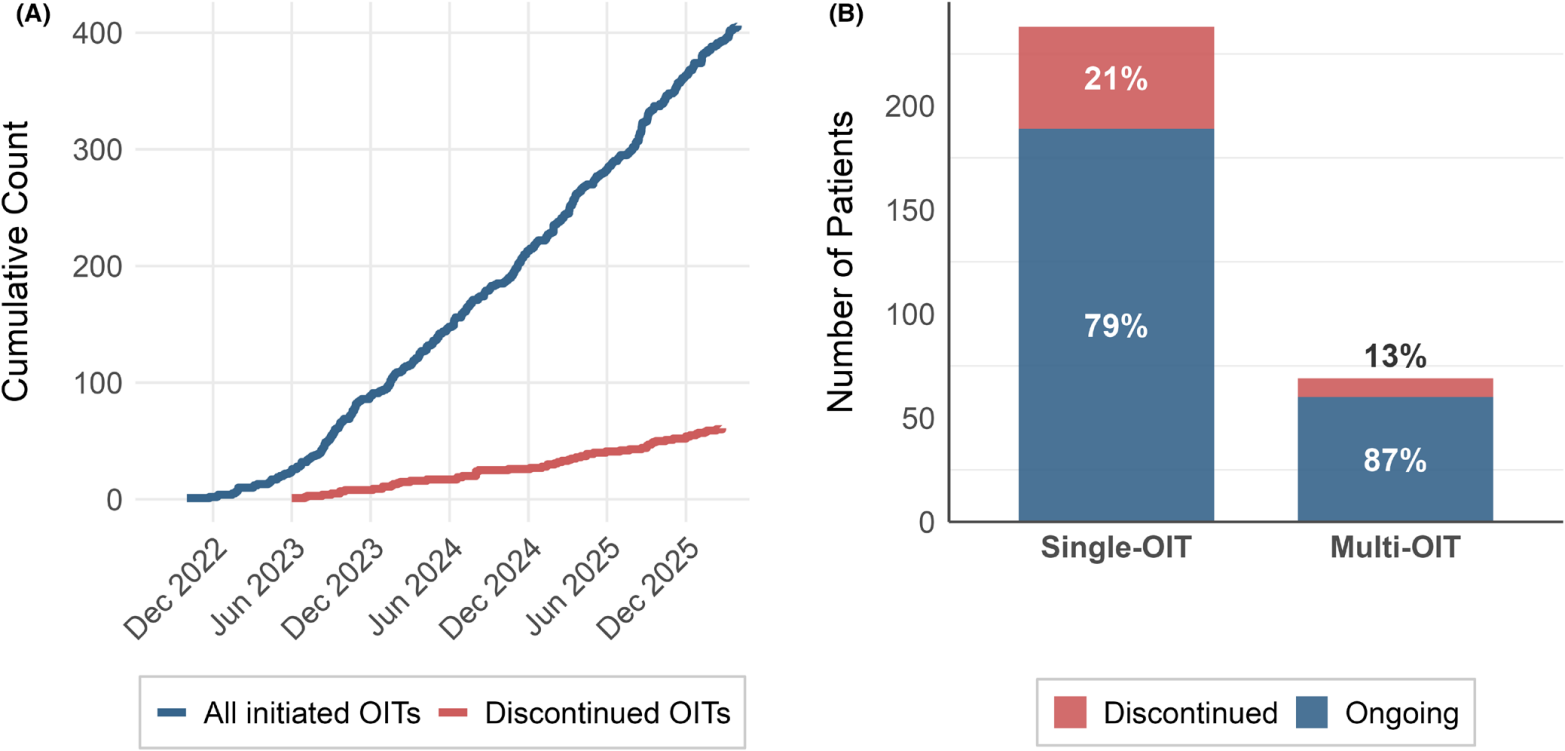
(A) Cumulative initiations (blue line) and discontinuation of oral immunotherapy (OIT, red line) from July 2022 to March 2026. (B) OIT discontinuation rates according to treatment complexity. Multi‐OIT indicates the treatment of two or more allergens. Absolute numbers (*n*) are represented by bar height, and relative frequencies (%) are displayed within the segments.

Between July 1, 2022 and March 31, 2026, 325 patients initiated 430 OIT courses. After excluding 17 patients without consent (23 OITs) and one patient lost to follow‐up (1 OIT), 406 OIT courses in 307 patients were included in the final analysis (Figures 1 and 2A). Overall, 69 patients (22.5%) underwent multi‐food OIT (multi‐OIT). A total of 61 OIT courses (15%) in 58 patients were discontinued. Two patients discontinued two concurrent OIT courses simultaneously, and one patient discontinued a second treatment attempt for the same allergen.

Multi‐OIT was not associated with an increased risk of discontinuation and showed numerically lower rates compared to single‐allergen OIT (13% vs. 21%, Figure 2B). Among patients undergoing multi‐food OIT, two discontinued two OIT courses: one because of taste aversion to both allergens and one because of EoE following peanut OIT and subsequent taste aversion to walnut. Among seven patients who discontinued only one of several OIT courses, five discontinuations were due to allergen‐specific adverse events, one to allergen‐specific taste aversion, and one to low motivation regarding the remaining OIT course.

In the discontinuation group, median age at initiation was 7 years (range: 0.7–19 years), and 31% had a history of severe systemic reactions prior to the OFC. Atopic dermatitis was the most common comorbidity (67%), followed by allergic rhinitis (47%) and asthma (28%) (Table 1). No significant differences were observed between the overall cohort and the discontinuation group with regard to sex, age, baseline sIgE, atopic comorbidities, or OIT initiation parameters including RD, OFC reaction severity, starting dose, and ratio of starting dose to RD (see Table S2).

**TABLE 1:** Clinical and immunological characteristics of the discontinuation group.

Patient characteristics	*N*	Overall	Adverse events	Taste aversion	Logistical issues	Anxiety and distress	*p*‐Value^a^
		*N* = 58^b^	*N* = 23^b^	*N* = 23^b^	*N* = 7^b^	*N* = 5^b^	
Sex (Female)	58	24 (41%)	9 (39%)	9 (39%)	3 (43%)	3 (60%)	.93
Asthma	58	16 (28%)	8 (35%)	4 (17%)	2 (29%)	2 (40%)	.53
Allergic Rhinitis	58	27 (47%)	12 (52%)	9 (39%)	3 (43%)	3 (60%)	.83
Atopic Dermatitis	58	39 (67%)	17 (74%)	13 (57%)	6 (86%)	3 (60%)	.42
Therapy Complexity (Multi‐OIT)	58	9 (16%)	5 (22%)	3 (13%)	1 (14%)	0 (0%)	.85

Treatment parameters	*N*	*N* = 61^c^	*N* = 25^c^	*N* = 24^c^	*N* = 7^c^	*N* = 5^c^	
Age at Start (years)	61	7.0 (5.0, 9.6)	6.2 (5.1, 8.5)	6.5 (4.3, 8.6)	11.6 (8.1, 12.1)	8.1 (8.0, 9.7)	.08
sIgE Allergen (kU/L)	61	8 (1, 61)	39 (4, 120)	4 (1, 45)	1 (1, 2)	8 (4, 30)	.02
sIgE major component (kU/L)^d^, ^e^	55	5 (1, 62)	22 (4, 120)	2 (0, 39)	1 (1, 2)	5 (4, 25)	.04
History of Severe Reaction (pre‐OFC)	61	19 (31%)	8 (32%)	8 (33%)	0 (0%)	3 (60%)	.16
OFC Characteristics							
RD at initial OFC (mg protein)^d^	60	300 (30, 1000)	100 (25, 650)	650 (45, 3000)	1000 (100, 3000)	1000 (100, 1000)	.19
Severity of reaction at initial OFC (oFASS‐5)^d^	60	2.00 (2.00, 3.00)	2.00 (2.00, 3.00)	3.00 (2.00, 3.00)	3.00 (2.00, 3.00)	3.00 (2.00, 3.00)	.54
OIT Characteristics							
Unplanned OIT^d^	60	18 (30%)	5 (21%)	9 (38%)	4 (57%)	0 (0%)	.12
Start Dose (mg protein)	61	80 (10, 300)	20 (6, 120)	100 (26, 300)	120 (20, 1000)	120 (40, 300)	.25
Ratio Start Dose to RD^d^	60	0.27 (0.12, 0.33)	0.27 (0.13, 0.33)	0.28 (0.11, 0.37)	0.15 (0.10, 0.33)	0.20 (0.12, 0.30)	.66
Duration until Discontinuation (months)	61	3.5 (1.0, 8.0)	2.1 (0.7, 4.2)	5.0 (1.7, 9.5)	7.4 (2.9, 29.7)	1.3 (0.8, 2.1)	.02
Dose before Discontinuation (mg protein)	61	300 (80, 1000)	80 (10, 500)	350 (270, 1000)	1000 (300, 1000)	300 (120, 300)	.009

*Note*: Patient‐level characteristics were analyzed once per patient. Treatment‐specific characteristics were analyzed per OIT course; therefore, patients discontinuing more than one OIT course could contribute more than one observation to the Treatment‐level section.

Abbreviations: oFASS‐5, ordinal Food Allergy Severity Scale; OFC, oral food challenge; OIT, oral immunotherapy; RD, reactive dose; sIgE, specific IgE.

^a^ Global comparison across all four discontinuation subgroups, Fisher's exact test or Kruskal–Wallis test.

^b^ Median (IQR) or Frequency (%); *N* represents the number of patients for demographic characteristics.

^c^ Median (IQR) or Frequency (%); *N* represents the total number of OIT courses for treatment‐specific parameters. Discrepancy due to two patients discontinuing two OITs each and one patient with two failed attempts for the same allergen.

^d^ Missing sIgEs to components in *n* = 6, missing variables for OFC parameters in *n* = 1 (only had an OFC at first OIT attempt).

^e^ Ara h 2, Cor a 14, Jug r 1, Ana o 3, Bos d 8/Casein, Ses i 1, Gly m 5, Gliadin, Gal d 1/Ovomucoid.

Four patients restarted OIT after a treatment gap of 3 months or more. Two had previously discontinued due to adverse events and restarted under omalizumab cover, with successful treatment achieved in one case. Two patients who had discontinued due to taste aversion successfully completed OIT upon re‐initiation at an older age.

### 3.2 Subgroup analysis of the discontinuation group

Baseline sIgE levels differed significantly across discontinuation subgroups (*p* = .02). Patients discontinuing due to AE had markedly higher sIgE levels (median 39 kU/L) compared to those discontinuing due to taste aversion, logistical barriers, or anxiety (median 4, 1, 8 kU/L, Table 1).

Age at OIT initiation also differed between subgroups (*p* = .08). Patients discontinuing due to logistical barriers or anxiety/distress were older (median 11.6 and 8.1 years, respectively) than those discontinuing due to AE or taste aversion (median 6.2 and 6.5 years) (Table 1, see Figure S1).

No significant differences were observed across subgroups for sex, atopic comorbidities, or baseline OFC parameters including reaction severity. However, patients in the AE subgroup showed a trend toward lower RD at baseline OFC (median 100 mg protein) and lower OIT starting doses (median 20 mg protein), consistent with a higher‐sensitivity phenotype. In contrast, patients discontinuing due to logistical barriers more frequently initiated therapy on an ad‐hoc basis directly following their baseline OFC (57%) and had higher RD (median 1000 mg protein) (Table 1).

### 3.3 Primary reasons for discontinuation

AE and taste aversion were the most frequent reasons for discontinuation (41% and 39%, respectively), followed by logistical barriers (11%) and anxiety/distress (8%) (Figure 1).

Within the AE group, 60% of discontinuations were due to immediate reactions, which were predominantly of moderate severity (oFASS‐5 grade 2–3). Among these, persistent gastrointestinal symptoms following dose administration were the most common manifestation, affecting six of eight patients. Severe immediate reactions (oFASS‐5 grade 4–5) led to discontinuation in six patients; four reactions occurred during home dosing, two of which required adrenaline administration. One severe reaction during the up‐dosing phase was complicated by supraventricular tachycardia following adrenaline administration (see Table S3).

Non‐immediate reactions accounted for 40% of AE‐related discontinuations and were predominantly EoE‐like in character (50% of non‐immediate cases, Figure 1). The majority of affected patients declined endoscopic evaluation and opted to discontinue therapy; symptoms resolved in all cases following cessation. Biopsy‐confirmed EoE was diagnosed in one patient with peanut OIT. Additional non‐immediate manifestations included atopic dermatitis exacerbation and unexplained coughing after food intake (not limited to the allergen of interest). This patient notably failed two separate OIT attempts for milk, once with and once without omalizumab cover due to this persistent, non‐specific respiratory symptom.

### 3.4 Temporal patterns and dosing until OIT discontinuation

**FIGURE 3:**
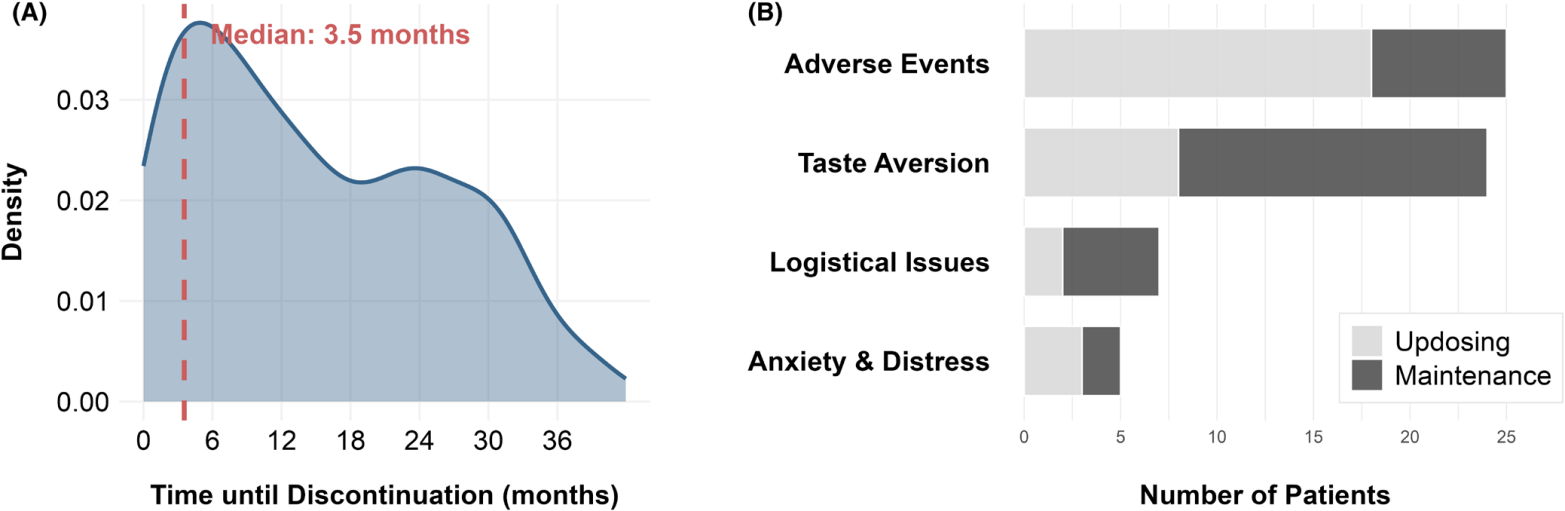
(A) Distribution of therapy duration until discontinuation. The density plot illustrates the timing of OIT attrition over the study period. The median time is indicated by the red dashed line. (B) Timing and primary reasons for OIT discontinuation. The bar chart illustrates the distribution of reasons for the premature termination of OIT, categorized by the treatment phase at the time of discontinuation. OIT, oral immunotherapy.

**FIGURE 4:**
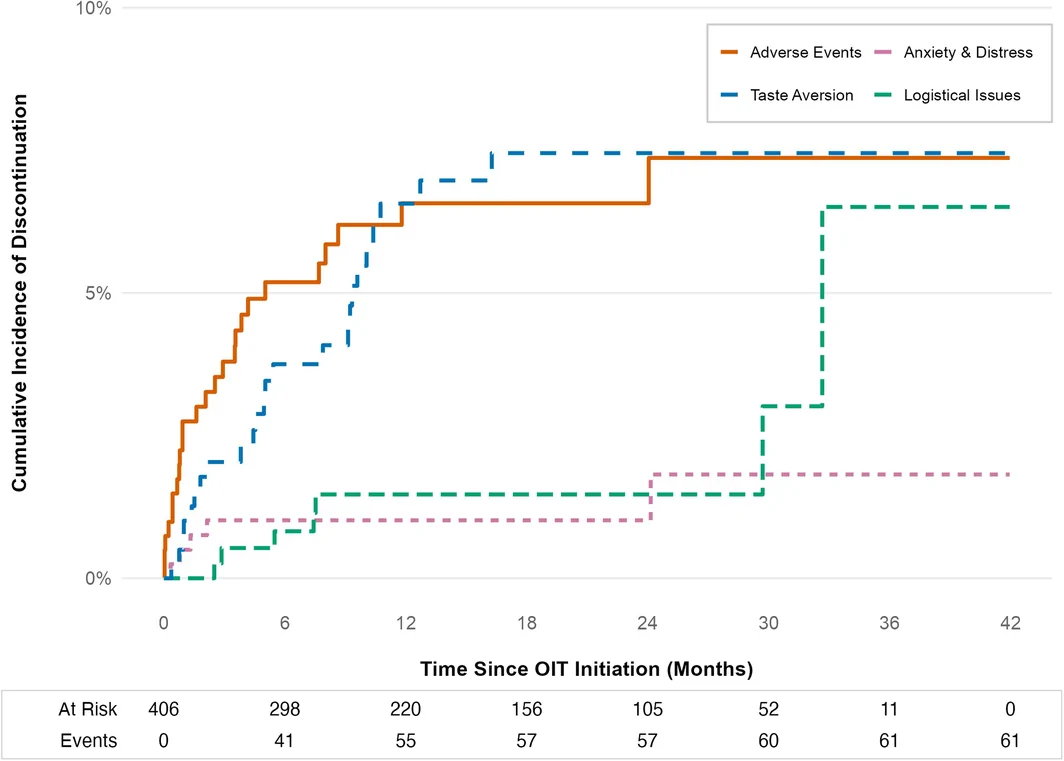
Cumulative incidence of OIT discontinuation by reason. Curves are shown for discontinuation reasons, with alternative reasons treated as competing events. Ongoing courses were censored at the study cut‐off on March 31, 2026. The risk table shows the number of OIT courses at risk and the cumulative number of events. Individual OIT courses were the unit of analysis; therefore, patients receiving OIT for more than one allergen could contribute more than one observation. OIT, oral immunotherapy.

Discontinuation occurred nearly equally during the up‐dosing phase (51%) and the maintenance phase (49%). Median time to discontinuation was 3.5 months (IQR 1–8) (Table 1, Figure 3A). The distribution of OIT duration for discontinued courses and observation duration for ongoing courses is shown in Figure S2. Timing differed markedly by reason for discontinuation, with distinct temporal patterns demonstrated in the competing‐risk analysis (Figure 3B, Figure 4). AE‐related dropout occurred earlier (median 2.1 months), while taste aversion was associated with later discontinuation (median 5 months), predominantly during the maintenance phase. The longest treatment duration prior to discontinuation was observed in the logistical barrier subgroup (median 7.4 months) (Table 1).

The median dose at the time of discontinuation was 300 mg protein (IQR 80–1000) and differed significantly across subgroups (*p* = .009). Patients discontinuing due to AE stopped at the lowest doses (median 80 mg). In contrast, patients discontinuing due to logistical barriers had reached the highest doses prior to cessation (median 1000 mg) (Table 1).

### 3.5 Subgroup analysis by allergen

Peanut was the most frequently treated allergen in the overall cohort, followed by cashew, hazelnut, and walnut. Potential observation time was similar for most major allergen groups, with median values ranging from 14.7 to 17.0 months for cashew, hazelnut, milk, peanut, and walnut. Sesame had a shorter median potential observation time of 7.3 months, whereas wheat had the longest at 28.0 months (Table S4).

**FIGURE 5:**
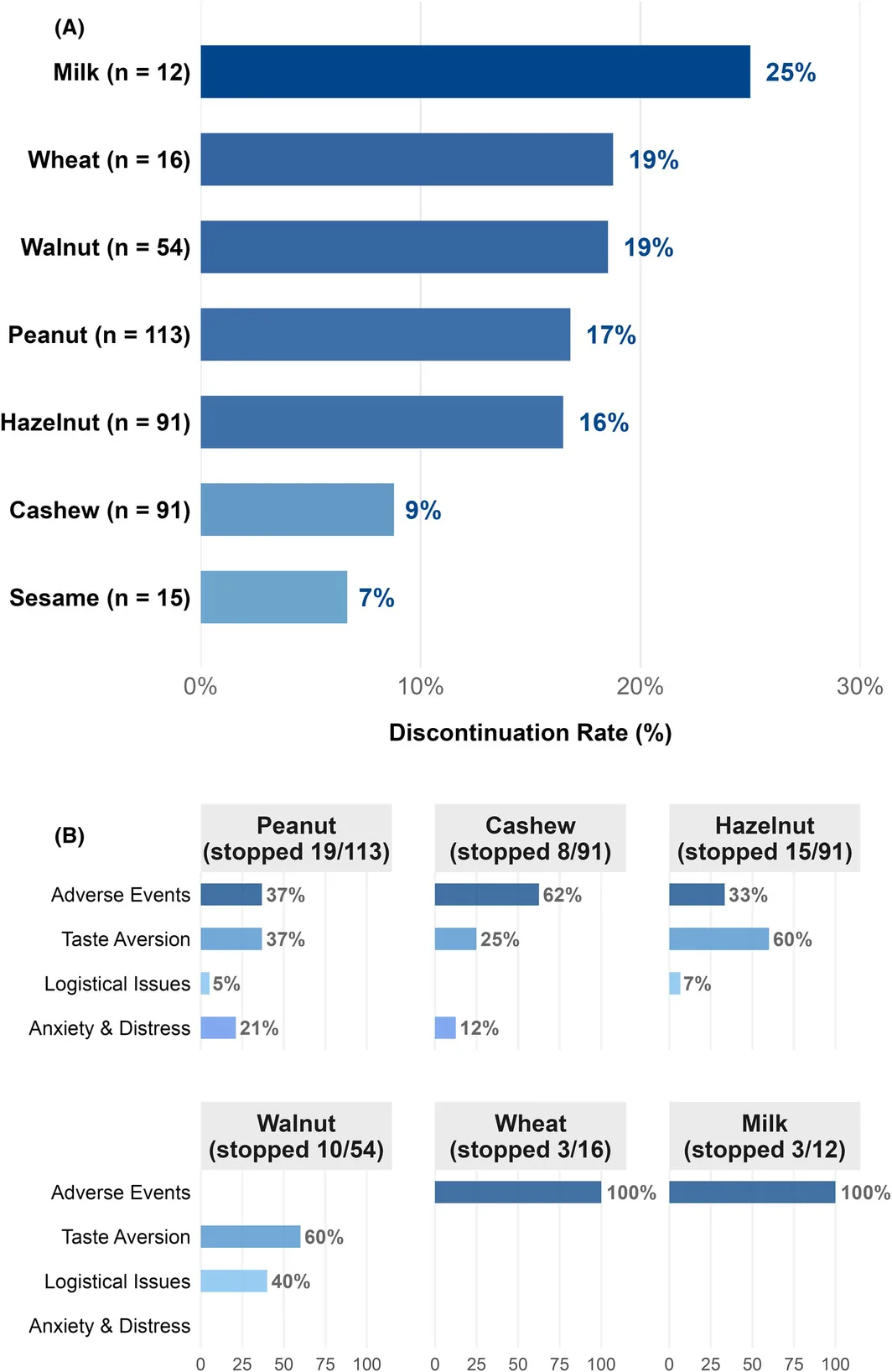
(A) Discontinuation rates per food allergen. Bars represent the percentage of discontinued treatment courses; absolute numbers of initiated oral immunotherapies (OIT) are indicated in parentheses next to each allergen label. (B) Primary reasons for discontinued OIT courses for each food. Bars represent the proportional distribution of discontinuation reasons.

Discontinuation rates varied by allergen and were highest for milk (25%), followed by wheat (19%), walnut (19%), peanut (17%), hazelnut (16%), cashew (9%), and sesame (7%) (Figure 5A).

Reasons for discontinuation differed by allergen (Figure 5B). Taste aversion accounted for the majority of discontinuations in the hazelnut and walnut subgroups, whereas AE were the predominant reason for discontinuations in the cashew, milk, and wheat subgroups. Most discontinuations attributed to anxiety and distress occurred in the peanut subgroup.

### 3.6 Cluster analysis

Hierarchical clustering identified three clusters (average silhouette width: 0.45; Jaccard indices: 0.88–0.97). Cluster 1 (*n* = 15) comprised older patients (median 11.2 years) with universal asthma comorbidity and a high rate of prior severe reactions (47%). Cluster 2 (*n* = 31) consisted of younger patients (median 6.9 years) with the lowest sIgE levels and no history of severe reactions. Cluster 3 (*n* = 11) was characterized by younger age (median 6.1 years), the highest sIgE levels, a universal history of severe reactions, and the lowest RD at baseline OFC (Table 2).

**TABLE 2:** Characterization of patient clusters based on clinical and immunological phenotypes.

Cluster	1. *N* = 15^b^	2. *N* = 31^b^	3. *N* = 11^b^	*p*‐Value^a^
Variables included in cluster analysis				
Age at OIT initiation (years)	11.2 (8.1, 15.1)	6.9 (4.3, 8.5)	6.1 (4.4, 7.0)	.001
sIgE level (log‐transformed, kU/L)	1.09 (0.11, 1.78)	0.34 (0.02, 1.08)	1.76 (0.66, 2.08)	.009
Asthma	15 (100%)	0 (0%)	0 (0%)	<.001
History of Severe Reaction (pre‐OFC)	7 (47%)	0 (0%)	11 (100%)	<.001
RD at initial OFC (mg Protein)	300 (100, 1000)	300 (60, 3000)	100 (30, 1000)	.41
Variables not included in cluster analysis				
Primary reason for discontinuation				
Taste Aversion	4 (27%)	13 (42%)	6 (55%)	.7
Adverse Events	7 (47%)	11 (35%)	4 (36%)	
Logistical Issues	2 (13%)	5 (16%)	0 (0%)	
Anxiety and Distress	2 (13%)	2 (6.5%)	1 (9.1%)	
OIT Allergen				
Peanut	4 (27%)	10 (32%)	4 (36%)	.11
Hazelnut	3 (20%)	8 (26%)	3 (27%)	
Cashew	3 (20%)	3 (9.7%)	2 (18%)	
Walnut	0 (0%)	9 (29%)	1 (9.1%)	
Sesame	1 (6.7%)	0 (0%)	0 (0%)	
Milk	1 (6.7%)	0 (0%)	0 (0%)	
Wheat	2 (13%)	1 (3.2%)	0 (0%)	
Macadamia	1 (6.7%)	0 (0%)	0 (0%)	
Soy	0 (0%)	0 (0%)	1 (9.1%)	

*Note*: Patient clusters were identified via hierarchical clustering using Gower's distance and Ward's minimum variance method (*k* = 3). Primary reasons for discontinuation were included as validation variables to assess the clinical outcomes of each cluster. Cases with missing baseline data were excluded from the analysis (*n* = 1).

Abbreviations: OFC, oral food challenge; OIT, oral immunotherapy; sIgE, specific IgE; RD, reactive dose.

^a^ Kruskal–Wallis rank sum test; Fisher's exact test.

^b^ Median (Q1, Q3); *n* (%).

Baseline characteristics differed significantly across clusters (Table 2), whereas neither the distribution of OIT allergens (*p* = .11) nor the primary reason for discontinuation (*p* = .70) differed significantly. However, the distribution of reasons varied between groups. Taste aversion was the most frequent reason for discontinuation in Cluster 3 (55%) and Cluster 2 (42%), whereas AE were the most frequent reason in Cluster 1 (47%) (Table 2).

### 3.7 Concomitant biologic therapy during OIT

Eight patients received concomitant omalizumab during OIT (Table S2). Indications included high‐risk OIT initiation (*n* = 3), rescue therapy following anaphylaxis during up‐dosing (*n* = 2), and re‐initiation of OIT after prior discontinuation due to adverse events (*n* = 2; successful in one case). In one of these patients, omalizumab had to be continued beyond the maintenance phase due to recurrent reactions upon attempted withdrawal. One additional patient with EoE in remission under dupilumab was successfully desensitized to wheat, with sustained EoE remission.

## 4 DISCUSSION

This study provides one of the first real‐world analyses of OIT discontinuation across a broad spectrum of food allergens in a prospective pediatric cohort. The overall discontinuation rate was 15%, driven almost equally by adverse events and non‐AE factors, with taste aversion emerging as an important behavioral barrier to long‐term adherence. Exploratory cluster analysis identified potentially distinct clinical phenotypes with differing discontinuation patterns, offering a basis for more individualized patient monitoring and support.

### 4.1 Discontinuation rates and timing

The overall discontinuation rate of 15% aligns with previous real‐world data,^21^ but is lower than rates reported in large controlled trials.^7^, ^9^, ^27^ This discrepancy likely reflects the flexibility inherent to routine clinical practice, where protocols can be continuously adapted to individual patient tolerability. Allergen‐specific rates varied considerably, with milk showing the highest rate and cashew and sesame the lowest, consistent with prior reports.^28^ The low discontinuation rate for cashew is particularly noteworthy given the rising prevalence of cashew allergy in our region.^29^ The low rate observed for sesame should, however, be interpreted cautiously. Although OIT for all major allergens was introduced at our center within a similar period, sesame OIT was used more frequently only later during the study period and consequently had the shortest median potential observation time.

A distinctive finding of this cohort was that 49% of discontinuations occurred during the maintenance phase, later in the treatment course than reported elsewhere.^21^, ^30^ The median dose at discontinuation was 300 mg protein, a threshold generally regarded as protective against accidental exposure‐related severe reactions.^31^ These findings indicate that a substantial proportion of patients who discontinued had achieved a clinically relevant level of desensitization yet elected to stop, underscoring that immunological success does not guarantee long‐term adherence. These observations emphasize the need for continued structured follow‐up and motivational support well beyond the up‐dosing phase.

Multi‐food OIT was not associated with increased discontinuation; if anything, discontinuation rates were lower than for single‐allergen OIT. Whether this reflects a selection effect, whereby patients pursuing multi‐food OIT are more highly motivated, or a genuine treatment‐related phenomenon warrants further evaluation.

### 4.2 The challenge of taste aversion

Taste aversion was one of the most frequent reasons for discontinuation and was the predominant driver of dropout in the hazelnut and walnut subgroups. Although taste aversion has been described primarily in older children,^20^, ^32^ it was equally prevalent in younger patients in this cohort. This is clinically important given the growing interest in early‐intervention OIT, where high motivation and treatment acceptance are often assumed.^7^, ^14^ The persistence of taste aversion into the maintenance phase in most affected patients suggests that sensory intolerance does not reliably resolve with continued exposure and that discontinuation due to taste aversion frequently occurs after a clinically meaningful degree of desensitization has already been achieved.

The occurrence of taste aversion as the predominant reason for stopping even in the highest‐risk immunological cluster, characterized by elevated sIgE, universal history of severe reactions, and low reactive dose, indicates that behavioral barriers operate independently of biological risk profile and must be addressed proactively. Strategies to mitigate sensory barriers include dose vehicle modifications and low‐dose maintenance approaches, which may improve long‐term tolerability in sensitized individuals. Alternative delivery routes, including sublingual and epicutaneous immunotherapy, offer a potential solution for patients in whom sensorial properties of the allergen represent an insurmontable barrier to oral dosing, though head‐to‐head comparative data on adherence outcomes relative to OIT remain limited.^33^, ^34^ Increasing child autonomy and stronger food preferences with age may contribute to the persistence of taste aversion. Previous patterns of strict allergen avoidance and parental anxiety around food exposure could also plausibly influence acceptance of repeated allergen ingestion during OIT^35^; however, these factors were not systematically captured in our cohort and could not be evaluated. Early and systematic identification of sensory barriers at treatment initiation, combined with individualized formulation strategies, should be integrated into future OIT protocols.

### 4.3 Adverse events and clinical phenotypes

Adverse events led to discontinuation in 41% of cases, predominantly due to moderate immediate reactions occurring early in the up‐dosing phase. Compared to some controlled trials and real‐world cohorts,^7^, ^16^, ^19^, ^22^ the AE‐related dropout was relatively infrequent in our cohort, which may reflect proactive protocol adaptations including dose reductions and extended up‐dosing intervals in patients with recurrent reactions. Anaphylaxis is a recognized risk of OIT,^6^, ^15^ particularly in the presence of co‐factors such as exercise, intercurrent illness, or hormonal variation,^17^, ^36^ and reported risk factors include older age, elevated immunological parameters, and atopic comorbidities.^23^, ^37^ Consistent with this, patients discontinuing due to AE in our cohort had significantly higher baseline sIgE levels, and the lower starting doses and reactive doses in this subgroup further support a higher‐sensitivity phenotype. While individual risk factors were not independently associated with discontinuation, exploratory cluster analysis suggested that their combination may be informative: the phenotype defined by older age, comorbid asthma, and elevated sIgE (Cluster 1) showed a trend toward more AE‐related dropouts, supporting the concept that multivariable risk profiling is more useful than single‐parameter assessment for patient stratification.

### 4.4 Psychological and logistical factors

Anxiety and distress accounted as the primary reason for discontinuation in 8% of cases, though the true prevalence is likely higher given the challenges of distinguishing anticipatory anxiety from somatic allergic symptoms in routine clinical practice.^20^ The concentration of almost all anxiety‐related discontinuations within the peanut subgroup is consistent with the well‐described heightened fear response associated with peanut allergy specifically. Standardized psychological screening is not currently integrated into our clinical pathway, and its absence represents a gap that future protocols should address through structured assessment at treatment initiation and key transition points such as the start of the maintenance phase. Logistical barriers were more prevalent in older patients with higher reactive doses, a pattern that may reflect diminishing perceived treatment necessity as baseline risk appears lower in this group. Unlike other cohorts,^20^, ^21^ socioeconomic factors were not identified as a relevant driver of discontinuation in our study, likely reflecting the broad insurance coverage for OIT within the Swiss healthcare system.

### 4.5 Strengths and limitations

This study benefits from prospective data collection using a standardized institutional database, a broad allergen spectrum, and a relatively large cohort for a single‐center real‐world study, enabling allergen‐specific and phenotype‐level analyses not possible in most prior reports. Importantly, the vast majority of patients initiating OIT at our center during the study period were enrolled (94%), with only 17 patients excluded due to absent consent and one lost to follow‐up. This near‐complete capture of the clinical population ensures that the findings reflect real‐world practice rather than a selected subset, minimizing the ascertainment bias that affects studies with lower enrollment rates. Limitations include the single‐center design, which may limit generalizability, and smaller subgroup sizes for less common allergens. The classification of a single primary reason for discontinuation was chosen to provide a clear analytical focus and in our clinical experience, one factor usually predominates in the decision to stop OIT. However, this approach simplifies what is often a multifactorial decision, and concurrent or evolving contributing factors may be underrepresented, potentially leading to misclassification. Differences between discontinuation categories should therefore be interpreted with this limitation in mind. The cluster analysis was exploratory and based on a relatively small discontinuation cohort. Because Gower distance assigns comparable contributions to variables of different types, the binary variables asthma and history of severe reactions may have disproportionately influenced the observed separation. The identified clusters should therefore be interpreted as hypothesis‐generating patterns. Finally, a proportion of ongoing OIT courses remain in early treatment phases at data cut‐off, which may lead to underestimation of long‐term discontinuation rates.

## 5 CONCLUSION

In this prospective real‐world cohort spanning a broad allergen spectrum, OIT discontinuation was driven equally by adverse events and behavioral barriers, with taste aversion representing a central and underappreciated threat to long‐term treatment success, including in young children and patients with the highest immunological risk. Importantly, adverse events and taste aversion represented distinct pathways to discontinuation, differing in timing and opportunities for intervention. Exploratory clustering suggested differing clinical phenotypes and discontinuation profiles based on routinely available baseline parameters, providing hypotheses for future risk‐adapted monitoring strategies in clinical practice. Taken together, these findings underscore that immunological desensitization is necessary but not sufficient for sustained OIT success: a substantial proportion of patients who discontinued had already achieved protective allergen thresholds yet elected to stop. Improving real‐world OIT outcomes will require patient‐centered protocols that systematically address sensory, psychological, and logistical barriers alongside immunological risk from treatment initiation through the maintenance phase and beyond.

## AUTHOR CONTRIBUTIONS

**Boucq Camille:** Writing – review and editing; investigation. **Höfer Veronika:** Data curation; validation; writing – review and editing; project administration. **Trück Johannes:** Conceptualization; writing – review and editing; supervision; methodology; project administration; resources. **Breiding Maria:** Conceptualization; writing – original draft; formal analysis; methodology; investigation; visualization; writing – review and editing.

## FUNDING INFORMATION

This research did not receive any specific grant from funding agencies in the public, commercial, or not‐for‐profit sectors.

## CONFLICT OF INTEREST STATEMENT

The authors declare no conflicts of interest related to this manuscript.

## Supporting information


Table S1.

## Data Availability

The data that support the findings of this study are available on request from the corresponding author. The data are not publicly available due to privacy or ethical restrictions.

## References

[1] Islam N , Chu AWL , Sheriff F , et al. Risk factors for the development of food allergy in infants and children: a systematic review and meta‐analysis. JAMA Pediatr. 2026;180:486‐499. doi:10.1001/jamapediatrics.2025.6105 41661638 PMC12887841

[2] Grabenhenrich LB , Dölle S , Moneret‐Vautrin A , et al. Anaphylaxis in children and adolescents: the European anaphylaxis registry. J Allergy Clin Immunol. 2016;137(4):1128‐1137.e1. doi:10.1016/j.jaci.2015.11.015 26806049

[3] Sicherer SH , Noone SA , Muñoz‐Furlong A . The impact of childhood food allergy on quality of life. Ann Allergy Asthma Immunol. 2001;87(6):461‐464. doi:10.1016/S1081-1206(10)62258-2 11770692

[4] Stensgaard A , Bindslev‐Jensen C , Nielsen D , Munch M , DunnGalvin A . Quality of life in childhood, adolescence and adult food allergy: patient and parent perspectives. Clin Exp Allergy. 2017;47(4):530‐539. doi:10.1111/cea.12849 27976436

[5] Santos AF , Riggioni C , Agache I , et al. EAACI guidelines on the management of IgE‐mediated food allergy. Allergy. 2025;80(1):14‐36. doi:10.1111/all.16345 39473345 PMC11724237

[6] The PALISADE Group of Clinical Investigators . AR101 Oral immunotherapy for Peanut allergy. N Engl J Med. 2018;379(21):1991‐2001. doi:10.1056/NEJMoa1812856 30449234

[7] Jones SM , Kim EH , Nadeau KC , et al. Efficacy and safety of oral immunotherapy in children aged 1–3 years with peanut allergy (the immune tolerance network IMPACT trial): a randomised placebo‐controlled study. Lancet. 2022;399(10322):359‐371. doi:10.1016/S0140-6736(21)02390-4 35065784 PMC9119642

[8] Riggioni C , Oton T , Carmona L , Du Toit G , Skypala I , Santos AF . Immunotherapy and biologics in the management of ige ‐mediated food allergy: systematic review and meta‐analyses of efficacy and safety. Allergy. 2024;79(8):2097‐2127. doi:10.1111/all.16129 38747333

[9] Chinthrajah RS , Purington N , Andorf S , et al. Sustained outcomes in oral immunotherapy for peanut allergy (POISED study): a large, randomised, double‐blind, placebo‐controlled, phase 2 study. Lancet. 2019;394(10207):1437‐1449. doi:10.1016/S0140-6736(19)31793-3 31522849 PMC6903389

[10] Michaud E , Amat F , Lambert C , et al. Real‐life safety of peanut oral immunotherapy: results from a French multicenter observational study. J Allergy Clin Immunol Glob. 2026;5(2):100628. doi:10.1016/j.jacig.2025.100628 41561977 PMC12814055

[11] Sabouraud M , Biermé P , André‐Gomez SA , et al. Real‐life experience with hazelnut oral immunotherapy. Ann Allergy Asthma Immunol. 2022;128(4):432‐438. doi:10.1016/j.anai.2022.01.002 35007745

[12] Kulmala P , Pelkonen AS , Kuitunen M , et al. Wheat oral immunotherapy was moderately successful but was associated with very frequent adverse events in children aged 6–18 years. Acta Paediatrica. 2018;107(5):861‐870. doi:10.1111/apa.14226 29345001

[13] Wasserman RL , Hague AR , Pence DM , et al. Real‐world experience with Peanut Oral immunotherapy: lessons learned from 270 patients. J Allergy Clin Immunol Pract. 2019;7(2):418‐426.e4. doi:10.1016/j.jaip.2018.05.023 29859333

[14] Vickery BP , Berglund JP , Burk CM , et al. Early oral immunotherapy in peanut‐allergic preschool children is safe and highly effective. J Allergy Clin Immunol. 2017;139(1):173‐181.e8. doi:10.1016/j.jaci.2016.05.027 27522159 PMC5222765

[15] Chu DK , Wood RA , French S , et al. Oral immunotherapy for peanut allergy (PACE): a systematic review and meta‐analysis of efficacy and safety. Lancet. 2019;393(10187):2222‐2232. doi:10.1016/S0140-6736(19)30420-9 31030987

[16] Mori F , Cianferoni A , Brambilla A , et al. Side effects and their impact on the success of milk oral immunotherapy (OIT) in children. Int J Immun Pharm. 2017;30(2):182‐187. doi:10.1177/0394632017697986 PMC5806791 28466667

[17] Patel N , Vazquez‐Ortiz M , Turner PJ . Risk factors for adverse reactions during OIT. Curr Treat Opt Allergy. 2019;6(2):164‐174. doi:10.1007/s40521-019-00205-2

[18] Rossi CM , Terreehorst I , Apostolidou E , et al. A systematic review and meta‐analysis on the induction of confirmed eosinophilic esophagitis as a side effect of allergen immunotherapy: an eaaci task force report. Allergy. 2025;81:1024‐1038. doi:10.1111/all.70183 41403138 PMC13040641

[19] Vickery BP , Scurlock AM , Steele P , et al. Early and persistent gastrointestinal side effects predict withdrawal from Peanut Oral immunotherapy (OIT). J Allergy Clin Immunol. 2011;127(2):AB26. doi:10.1016/j.jaci.2010.12.114

[20] Trevisonno J , Venter C , Pickett‐Nairne K , et al. Age‐related food aversion and anxiety represent primary patient barriers to food Oral immunotherapy. J Allergy Clin Immunol Pract. 2024;12(7):1809‐1818.e3. doi:10.1016/j.jaip.2024.03.014 38492666

[21] Plessis AA , Cameron SB , Invik R , Hanna M , Mack DP , Cook VE . Real‐world experience: a retrospective pediatric chart review to determine why patients and caregivers discontinue oral immunotherapy. Allergy Asthma Clin Immunol. 2024;20(1):54. doi:10.1186/s13223-024-00912-9 39407324 PMC11481366

[22] Loke P , Orsini F , Lozinsky AC , et al. Probiotic peanut oral immunotherapy versus oral immunotherapy and placebo in children with peanut allergy in Australia (PPOIT‐003): a multicentre, randomised, phase 2b trial. Lancet Child Adolesc Health. 2022;6(3):171‐184. doi:10.1016/S2352-4642(22)00006-2 35123664

[23] Karunakaran D , Chan ES , Zhang Q , et al. Risk factors associated with safety of preschool peanut oral immunotherapy. J Allergy Clin Immunol Glob. 2023;2(2):100094. doi:10.1016/j.jacig.2023.100094 37780798 PMC10510002

[24] Fernández‐Rivas M , Gómez García I , Gonzalo‐Fernández A , et al. Development and validation of the food allergy severity score. Allergy. 2022;77(5):1545‐1558. doi:10.1111/all.15165 34716996 PMC9298738

[25] Breiding M , Höfer V , Kuhn A , et al. Predicting outcomes of hazelnut Oral food challenge: a pediatric cohort analysis. J Allergy Clin Immunol Glob. 2026;5:100688. doi:10.1016/j.jacig.2026.100688 42109933 PMC13157154

[26] Mueller HL . Diagnosis and treatment of insect sensitivity. J Asthma Res. 1966;3(4):331‐333. doi:10.3109/02770906609106941 4380730

[27] Lloyd M , Loke P , Nguyen K , et al. Probiotic oral immunotherapy for egg and milk allergy induces sustained unresponsiveness. Pediatric Allergy and Immunology. 2025;36(10):e70222. doi:10.1111/pai.70222 41097819

[28] Breiding M , Soomann M , Roth M , Trück J , Bellutti Enders F . Safety of oral immunotherapy for cashew nut and peanut allergy in children – a retrospective single‐centre study. Swiss Medical Weekly. 2024;154(11):3691. doi:10.57187/s.3691 39570923

[29] Höfer V , Dölle‐Bierke S , Sabouraud‐Leclerc D , et al. A growing concern for cashew and an unexpected risk from almonds: data from the anaphylaxis registry. Allergy. 2025;80:2837‐2848. doi:10.1111/all.16619 40511587 PMC12486339

[30] Soller L , Abrams EM , Carr S , et al. First real‐world safety analysis of preschool Peanut Oral immunotherapy. J Allergy Clin Immunol Pract. 2019;7(8):2759‐2767.e5. doi:10.1016/j.jaip.2019.04.010 31002957

[31] Baumert JL , Taylor SL , Koppelman SJ . Quantitative assessment of the safety benefits associated with increasing clinical Peanut thresholds through immunotherapy. J Allergy Clin Immunol Pract. 2018;6(2):457‐465.e4. doi:10.1016/j.jaip.2017.05.006 28669889

[32] Mack DP , Greenhawt M , Turner PJ , et al. Information needs of patients considering oral immunotherapy for food allergy. Clin Exp Allergy. 2022;52(12):1391‐1402. doi:10.1111/cea.14225 36083693

[33] Upton JEM , Toscano‐Rivero D , Ke D , et al. Peanut Oral immunotherapy using 30 and 300 mg maintenance doses. J Allergy Clin Immunol Pract. 2026;14(1):223‐232.e7. doi:10.1016/j.jaip.2025.10.007 41109568

[34] Upton JEM , Li CH , Berenjy A , et al. Safety and efficacy of very low‐dose multi‐nut Oral immunotherapy in children. Clin Transl Allergy. 2025;15(12):e70125. doi:10.1002/clt2.70125 41385738 PMC12700494

[35] Mack DP , Dribin TE , Turner PJ , et al. Preparing patients for Oral immunotherapy (PPOINT): international Delphi consensus for procedural preparation and consent. J Allergy Clin Immunol. 2024;153(6):1621‐1633. doi:10.1016/j.jaci.2024.02.019 38597862 PMC11461787

[36] Afinogenova Y , Rubin TN , Patel SD , et al. Community private practice clinical experience with Peanut Oral immunotherapy. J Allergy Clin Immunol Pract. 2020;8(8):2727‐2735. doi:10.1016/j.jaip.2020.03.016 32247684

[37] Lloyd M , Ashley S , Chawla SS , et al. Impact of participant baseline factors on exposure‐adjusted incidence of treatment‐related adverse events during Peanut Oral immunotherapy. J Allergy Clin Immunol Pract. 2025;13(12):3355‐3363.e3. doi:10.1016/j.jaip.2025.09.010 40976354

