# Supplementary material for: Taste aversion and adverse events contribute equally to OIT discontinuation in children: A real‐world multi‐allergen analysis
Source: Pediatr Allergy Immunol. 2026 Sep 16;37(9):e70482. doi: 10.1111/pai.70482 (PMC13580977; doi:10.1111/pai.70482)
Supplement: Supplementary file 1 [file PAI-37-e70482-s001.docx]

**Supplementary Material**

**Tables**

**Table S1:** Definitions and documented circumstances for primary reasons of OIT discontinuation. Each discontinued OIT course was classified according to the predominant documented reason for treatment cessation.

| **Primary Reason** | **Definition** | **Documented circumstances leading to discontinuation** |
| --- | --- | --- |
| Adverse Events  (n = 25 OITs) | Discontinuation primarily attributable to immediate or non-immediate symptoms considered related to OIT. | Severe AE requiring cessation (n = 1); Severe AE followed by patient/family decision not to pursue further adaptation (n = 5); Single moderate AE followed by discontinuation (n=3); Recurrent adverse events including EoE/EoE-like symptoms despite attempts to continue (n = 12), atypical adverse events considered potentially OIT-related (n = 4). |
| Taste Aversion  (n = 24 OITs) | Refusal to continue consuming the allergen. | Patient refused to eat the allergen, no AE, no anxiety (n = 21); Patient refused to eat hazelnut due to aversion, additionaly mild adverse events (n = 1); No motivation to continue treatment due to dislike of taste (n = 2). |
| Logistical Issues  (n = 7 OITs) | Discontinuation primarily related to treatment burden, including insufficient perceived benefit relative to the effort required. | Unrelated medical issues of the patient (n = 3);  Treatment burden exceeding perceived benefit (n = 3);  Parental disagreement following separation (n = 1). |
| Anxiety and Distress (n = 5 OITs) | Discontinuation primarily driven by fear, anticipatory anxiety, emotional distress associated with OIT. | Anxiety around multiple foods (n = 1);  Emotional stress/pressure associated with OIT (n = 2);  Anxiety related to possible reactions to OIT (n = 2). |

**Table S2:** Baseline clinical and immunological characteristics of all patients initiating OIT and stratified by OIT status. The discontinuation group includes patients who terminated at least one OIT course prematurely, whereas the ongoing group includes patients successfully continuing all initiated therapies. For patients undergoing multiple OITs simultaneously, the highest recorded sIgE value and reaction severity as well as the lowest reactive dose at baseline were used for patient-level analysis.

| **Characteristic^a^** | **N** | **Overall  N = 307** | **Discontinued  N = 58** | **Ongoing  N = 249** | ***P-Value*^b^** |
| --- | --- | --- | --- | --- | --- |
| **Sex (female)** | 307 | 123 (40%) | 24 (41%) | 99 (40%) | .82 |
| **Age at Start (years)** | 307 | 6.4 (4.1, 9.1) | 7.1 (5.0, 9.6) | 6.2 (3.7, 9.0) | .14 |
| **Number of OIT-Allergens** | 307 |  |  |  | .32 |
| *1* |  | 238 (78%) | 49 (84%) | 189 (76%) |  |
| *2* |  | 53 (17%) | 8 (14%) | 45 (18%) |  |
| *3+* |  | 16 (5.2%) | 1 (1.7%) | 15 (6.0%) |  |
| **sIgE Allergen (kU/L)** | 303 | 8 (2, 41) | 12 (1, 68) | 8 (2, 33) | .69 |
| **sIgE Major Component (kU/L)^c^** | 300 | 5 (1, 26) | 7 (1, 47) | 5 (1, 22) | .28 |
| **History of Severe Reaction (pre-OFC)** | 307 | 83 (27%) | 19 (33%) | 64 (26%) | .28 |
| **Allergic Rhinitis** | 307 | 148 (48%) | 27 (47%) | 121 (49%) | .78 |
| **Asthma** | 307 | 71 (23%) | 16 (28%) | 55 (22%) | .37 |
| **Atopic Dermatitis** | 307 | 204 (66%) | 39 (67%) | 165 (66%) | .89 |
| **RD of Initial OFC, mg Protein** | 304 | 300 (30, 1,000) | 300 (30, 1,000) | 300 (30, 1,000) | .71 |
| **OIT Start Dose, mg Protein** | 307 | 80 (20, 300) | 80 (12, 300) | 80 (20, 300) | .85 |
| **Ratio Start Dose to RD** | 304 | 0.27 (0.12, 0.33) | 0.28 (0.13, 0.33) | 0.27 (0.12, 0.33) | .53 |
| **Unplanned OIT^d^** | 306 | 81 (26%) | 16 (28%) | 65 (26%) | .83 |
| **Severity of Reaction at OFC (oFASS-5)** | 302 | 3.00 (2.00, 3.00) | 2.00 (2.00, 3.00) | 3.00 (2.00, 3.00) | .05 |
| **Omalizumab** | 307 | 8 (2.6%) | 3 (5.2%) | 5 (2.0%) | .18 |
| ^a^n (%); Median (Q1, Q3) | | | | | |
| ^b^Pearson's Chi-squared test; Wilcoxon rank sum test; Fisher's exact test | | | | | |
| ^c^Ara h 2, Cor a 14, Jug r 1, Ana o 3, Bos d 8/Casein, Ses i 1, Gly m 5, Gliadin, Gal d 1/Ovomucoid | | | | | |
| ^d^OIT started as an ad-hoc decision after OFC | | | | | |
| *oFASS-5, ordinal Food Allergy Severity Score; OFC, oral food challenge; OIT, oral immunotherapy; RD, reactive dose; sIgE, specific immunoglobulin E.* | | | | | |

**Table S3:** Patients discontinuing OIT due to severe adverse events (oFASS-5 score 4–5)

| **Age^1^** | **Allergen, dose^2^** | **Comorbidities** | **Timing (phase)** | **Reaction** | **Cofactor** | **Treatment** |
| --- | --- | --- | --- | --- | --- | --- |
| **6** | Milk, 1000 | Asthma, AD, ARC, other FA | Maintenance | Wheezing, dyspnea, loss of consciousness | Physical activity, viral infection | Adrenaline (i.m., i.v.), AH, CS, ICU |
| **3** | Hazelnut, 3 | AD | Up-dosing | Angioedema, stridor, dyspnea | None | AH, Salbutamol |
| **7** | Hazelnut, 300 | Asthma, AD, ARC, other FA | Maintenance | Urticaria, dyspnea, coughing | None | AH, CS |
| **4** | Peanut, 6 | ARC, other FA | Up-dosing | Wheezing, coughing, dyspnea, urticaria | ARC (trigger: animal dander) | Adrenaline (i.m.), AH |
| **5** | Wheat, 550 | Other FA | Up-dosing | Wheezing, angioedema, vomiting, SVT after i.m. adrenaline | Mild viral infection | Adrenaline (i.m.), Salbutamol, AH |
| **16** | Macadamia, 300 | AD, ARC, other FA | Maintenance | Angioedema, abdominal pain, dyspnea | Menstruation | AH, CS |
| ^1^ Age in years at reaction | | | | | | |
| ^2^Dose leading to reaction (mg protein) | | | | | | |
| *AD, atopic dermatitis; AH, antihistamine; ARC, allergic rhinoconjunctivitis; CS, corticosteroids; FA, food allergy; ICU, intensive care unit; i.m., intramuscular; i.v., intravenous; oFASS-5, ordinal Food Allergy Severity Score; OIT, oral immunotherapy; SVT, supraventricular tachycardia.* | | | | | | |

**Table S4:** **Potential observation time by food allergen.** Data are median (IQR) months from OIT initiation to March 31, 2026. Only allergens with at least 10 initiated OIT courses are shown. *OIT, oral immunotherapy; IQR, interquartile range.*

| **Food Allergen** | **Potential Observation Time, median (IQR), months** |
| --- | --- |
| Milk | 17.0 (6.8-28.6) |
| Wheat | 28.0 (16.9–31.7) |
| Walnut | 15.3 (8.1-22.8) |
| Peanut | 17.0 (6.9-27.4) |
| Hazelnut | 16.7 (9.9-28.7) |
| Cashew | 14.7 (7.5-24.3) |
| Sesame | 7.3 (4.8-24.7) |

**Figures**

**Figure S1**: Age distribution of patients at the start of oral immunotherapy (OIT) by primary reason for treatment discontinuation. Individual data points represent single OIT courses (n=61). Boxplots show the median and interquartile range (IQR).

**Figure S2:** Duration of OIT by current treatment status and reason for discontinuation. Each dot represents an individual OIT course. Duration was calculated from OIT initiation until discontinuation or, for ongoing courses, until the study cut-off date of March 31, 2026. Boxplots indicate the median and interquartile range. Ongoing courses are shown in blue and discontinued courses in red. *OIT, oral immunotherapy.*
